# Long-term exposure to fine particle matter and all-cause mortality and cause-specific mortality in Japan: the JPHC Study

**DOI:** 10.1186/s12889-022-12829-2

**Published:** 2022-03-08

**Authors:** Norie Sawada, Tomoki Nakaya, Saori Kashima, Takashi Yorifuji, Tomoya Hanibuchi, Hadrien Charvat, Taiki Yamaji, Motoki Iwasaki, Manami Inoue, Hiroyasu Iso, Shoichiro Tsugane

**Affiliations:** 1 Epidemiology and Prevention Group, Center for Public Health Sciences, National Cancer Center, 5-1-1 Tsukiji, Chuo-ku, 104-0045 Tokyo, Japan; 2 Graduate School of Environmental Studies, Tohoku University, Sendai, Japan; 3 Environmental Health Sciences Laboratory, Graduate School of Advanced Science and Engineering, Hiroshima University, Hiroshima, Japan; 4 Department of Epidemiology, Dentistry and Pharmaceutical Sciences, Okayama University Graduate School of Medicine, Okayama, Japan; 5 Public Health Graduate School of Medicine, Osaka University, Suita, Japan

**Keywords:** Particulate matter <2.5µg/m^3^ (PM_2.5_), Mortality, Low level exposure, Japan, Prospective study

## Abstract

**Background:**

Many epidemiological studies have reported the association between exposure to particulate matter and mortality, but long-term prospective studies from Asian populations are sparse. Furthermore, associations at low levels of air pollution are not well clarified. Here, we evaluated associations between long-term exposure to particulate matter <2.5 µg/m^3^ (PM_2.5_) and mortality in a Japanese cohort with a relatively low exposure level.

**Methods:**

The Japan Public Health Center-based Prospective Study (JPHC Study) is a prospective cohort study of men and women aged 40-69 years in 1990 who were followed up through 2013 for mortality. In this cohort of 87,385 subjects who did not move residence during follow-up, average PM_2.5_ levels from 1998 to 2013 by linkage with 1-km^2^ grids of PM_2.5_ concentration were assigned to the residential addresses of all participants. To avoid exposure misclassification, we additionally evaluated the association between 5-year (1998-2002) cumulative exposure level and mortality during the follow-up period from 2003 to 2013 in 79,078 subjects. Cox proportional hazards models were used to calculate the association of long-term exposure to PM_2.5_ on mortality, with adjustment for several individual confounding factors.

**Results:**

Average PM_2.5_ was 11.6 µg/m^3^. Average PM_2.5_ exposure was not associated with all-cause mortality or cancer and respiratory disease mortality. However, average PM_2.5_ was positively associated with mortality from cardiovascular disease (hazard ratio (HR) of 1.23 (95%CI=1.08-1.40) per 1-µg/m^3^ increase; in particular, HR in mortality from cerebrovascular disease was 1.34 (95%CI=1.11-1.61) per 1-µg/m^3^ increase. Additionally, these results using cumulative 5-year PM_2.5_ data were similar to those using average PM_2.5_ over 15 years.

**Conclusions:**

We found evidence for a positive association between PM_2.5_ exposure and mortality from cardiovascular disease in a Japanese population, even in an area with relatively low-level air pollution.

**Supplementary Information:**

The online version contains supplementary material available at 10.1186/s12889-022-12829-2.

## Background

Several prospective cohort studies have reported positive associations between exposure to air pollution, particularly fine particulate matter with a diameter of 2.5 μm or less (PM_2.5_), and mortality risk [1–15]. The International Agency for Research on Cancer (IARC) has identified outdoor PM_2.5_ as a Group I carcinogenic factor for lung cancer [16, 17]. Furthermore, PM_2.5_ exposure is concluded to be a “modifiable factor contributing to cardiovascular morbidity and mortality” by the American Heart Association writing group [18]. Additionally, a meta-analysis and systematic review recently reported that there is clear evidence that PM_2.5_ is associated with increased all-cause mortality, and mortality from cardiovascular disease, lung cancer and respiratory disease, based on around 20 cohort studies [3]. However, many of these studies were conducted in Western countries, primarily in North America and Europe. Although the meta-analysis reported a summary risk ratio for all-cause mortality per 10 µg/m^3^ of 1.07 (95% confidence interval (CI) 1.04-1.11) in the Western Pacific region, this was based on only three studies (Taiwan, China and Hong Kong) [3, 6, 7, 19]. Furthermore, the meta-analysis also suggested that PM_2.5_ is associated with increased risk for mortality at low exposure levels, even below the current WHO guideline exposure level of 10 µg/m^3^ [3].

Outdoor air pollution levels have decreased over the last few decades in developed countries, including America [20], Europe [21] and Japan [22]. This decline highlights the importance of examining the adverse effects of this pollution at low levels.

Our research group, The Japan Public Health Center-based Prospective Study (JPHC Study), reported in 2013 that particulate matter did not increase the risk of mortality of cardiovascular disease or lung cancer mortality in the Japanese population [23]. However, exposure level in each area in that study was derived from single area using the nearest monitoring station, meaning that nine exposure levels were assigned to nine areas. Accordingly, the study was unable to determine individual exposure levels.

Here, we have updated this JPHC study analysis by improving the assessment of PM _2.5_ exposure, from the one-point assessment in each study area to substantially higher resolution (approximately 1-km^2^ grids) and by limiting subjects to those who did not move during follow-up. Additionally, we were able to evaluate never smokers due to the extension of the follow-up period and the increased number of deaths in the cohort.

## Methods

### Study population

The study population was derived from the JPHC Study, a population-based cohort which consists of two separate cohorts (I and II) with a total of 140,420 study participants (68,722 men and 71,698 women). Details of the study design have been provided elsewhere [24, 25]. Cohort I was launched in 1990 and consists of 61,595 residents from 5 public health center areas, namely Yokote (Akita Prefecture), Ninohe (Iwate Prefecture), Katsushika (Tokyo), Saku (Nagano Prefecture) and Chubu (Okinawa Prefecture). Cohort II was started in 1993 and includes 78,825 residents from 6 public health center areas, Nagaoka (Niigata Prefecture), Mito (Ibaraki Prefecture), Suita (Osaka Prefecture), Kamigoto (Nagasaki Prefecture), Chuo-higashi (Kochi Prefecture) and Miyako (Okinawa Prefecture). All subjects were aged 40-59 years in Cohort I and 40-69 years in Cohort II at baseline, except in Tokyo-Katsushika and Osaka-Suita, where are located within major cities: in Tokyo-Katsushika, subjects were selected at the time of their 40- or 50-year health checkup, which were conducted by the Katsushika ward; while in the Osaka-Suita area, one group was drawn from residents aged 40 or 50 years who were invited to participate in a comprehensive health checkup program conducted by the city and a second group was randomly selected from the population registry of the city after stratification by sex and 10-year age group. The baseline survey was conducted from 1990 to 1994 in Cohort I and from 1993 to 1995 in Cohort II.

To minimize misclassification of exposure to air pollution, we limited assignment of PM_2.5_ levels to the 103,639 subjects (50,686 men, 52,953 women) who did not change residential address from that at baseline during follow-up. Among these, 87,645 (41,489 men, 46,156 women) responded to a questionnaire (response rate=84.6%) regarding lifestyle, diet, past history of diseases, etc. The study protocol conforms to the ethical guidelines of the Declaration of Helsinki and informed consent was obtained from all participants when completing the survey questionnaire. The study protocol including informed consent was approved by the Institutional Review Board of the National Cancer Center (Number 2001-021, 2015-085, 2016-154).

### Air pollution measurements

Ambient PM_2.5_ at the residential address of study participants was assigned through application of a global land use regression (LUR) model. Model construction is described in detail elsewhere [26]; briefly, annual satellite-derived measurements of PM_2.5_ were converted to near-ground concentrations via the GEOS-Chem transport model and a ground-based sun photometer. Estimates of PM_2.5_ were generated at a spatial scale of 1 km x 1 km (0.01°× 0.01° longitude-latitude) and found to correspond well with ground-based measurements (R^2^: 0.81) [26]. Exposure to PM_2.5_ was assigned as the average PM_2.5_ from 1998 to 2013 at the geocoded location of individual residences at baseline, among subjects who were confirmed not to have moved. We excluded 260 participants who had missing PM_2.5_ concentrations for any year during follow-up. After these exclusions, 87,385 subjects (41,362 males and 46,023 females) remained for final analysis.

We evaluated two types of PM_2.5_ concentration. The primary exposure variable used the average PM_2.5_ level over the period. We evaluated the association between average exposure level (from 1998 to 2013) and mortality in the 87,385 subjects during the follow-up period (1990-2013). This exposure may not have been appropriate due to a lack of data from 1990 to 1997, and this may have caused misclassification. To avoid this misclassification, we additionally created a second exposure variable - the cumulative PM_2.5_ level for 5 years (1998-2002) – with which we evaluated the association between 5-year cumulative exposure level and mortality during follow-up period from 2003 to 2013 in 79,078 subjects, after excluding subjects who were lost to follow-up or died before 2003.

### Follow-up and mortality

We followed all registered subjects from questionnaire response date to 31 December 2013. Changes in residence status were identified in each study area through the residential registry. After we identified the survival status of subjects, we linked records with vital statistics data from the Ministry of Health, Labor, and Welfare, with permission. Registration of deaths is mandatory under the Family Registration Law, and the registry is nearly 100% complete in Japan. Causes of death were coded according to the 10th International Classification of Diseases (ICD-10). For cause-specific mortality analyses, deaths from cancer (C00-C99), cardiovascular disease (I01-I99) and respiratory disease (J00-J99) were identified. Further, we also evaluated cause of death in more detail for lung cancer (C34), ischemic heart disease (IHD) (I20-I25), cerebrovascular disease (I60-I69), pneumonia and influenza (J10-J22) and chronic obstructive pulmonary disease (COPD) and associated conditions (J40-J47).

### Statistical analysis

Cox proportional hazards models were used to quantify the association between PM_2.5_ level and mortality. Hazard ratios (HRs) and 95% confidence intervals (CIs) were calculated for every 1-µg/m^3^ increase in PM_2.5_ exposure levels.

As primary analysis, we first analyzed the dataset (N = 87,385, 41,362 men and 46,023 women) for the follow-up period (days) 1990-2013 by using average annual PM_2.5_ levels from 1998 to 2013. Person-years of follow-up were calculated from the date of response to the baseline questionnaire to the end of follow-up (December 31st 2013), loss to follow-up or date of death, whichever occurred first. We first adjusted for sex, age (continuous) and public health center (PHC) (11 areas by strata) using the baseline questionnaire. We then further adjusted for confounding factors smoking status (never, past, current smokers (<20 cigarettes/day, ≧20 cigarettes /day), missing), alcohol drinking (never, occasional, <150 g ethanol/week, 150-300 g ethanol/week, ≧300 g ethanol/week, missing), body mass index (<18.5, 18.5-23, 23-25, ≧25, missing), occupation (primary, secondary, tertiary, housewife or unemployed, missing), coffee drinking (1 cup/day, 1-2 cups/day, 3-4 cups/day, ≧5 cups/day, missing), passive smoking at home/workplace (yes, no, missing), and a past history of cardiovascular disease (yes, no), cancer (yes, no) or diabetes mellitus (yes, no). Furthermore, we adjusted for birth year to control between-cohort effects.

Next, for sensitivity analysis, we analyzed a dataset (N = 79,078, 36,665 men and 42,413 women) for the follow-up period (days) from 2003 to 2013 by using 5-year cumulative PM_2.5_ levels (1998-2002). Person-years of follow-up were calculated from January 1st 2003 to the end of follow-up (December 31st 2013), loss to follow-up or date of death, whichever occurred first. We adjusted for the same kind of variables as in the primary analysis, data for which were collected in the 10-yr follow-up survey (in 2000 for Cohort I and in 2003 for Cohort II).

We also calculated the association between average PM_2.5_ (1998-2003) and cumulative PM_2.5_ (5-year) levels and mortality in never smokers. Whenever a significant association was found between PM_2.5_ (1998-2003) or cumulative PM_2.5_ (5-year) levels and the mortality outcomes under study, we assessed the potential non-linearity of this relationship through a likelihood ratio test comparing the current model (i.e., including a linear effect of the PM_2.5_ variable) and a model in which the effect of the variable was modelled by a restricted cubic spline with a knot placed at the median of its distribution. All p values were two-sided. Analyses were conducted using the PHREG command of the SAS software (version 9.3; SAS Institute Inc., Cary, NC, USA), as well as the ‘survival’ and ‘spline’ R packages (R statistical software version 4.1.0; R Development Core Team, 2021).

## Results

The baseline characteristics of total subjects and never smokers are listed in Table 1. Annual average PM_2.5_ exposure (±Standard Deviation) was 11.6 (±2.9) µg/m^3^. The interquartile range was from 9.24 to 12.98 and the minimum and maximum were 7.18 and 17.89 µg/m^3^, respectively. Among total subjects, mean age (±standard deviation) was 51.9 (±7.9) and ever smokers accounted for 40.3%. For never smokers, the proportion of women was 81.3%, while that of regular drinkers, coffee drinkers, past history of diabetes mellitus, cardiovascular diseases and cancer were less compared with total subjects.

**Table 1:**
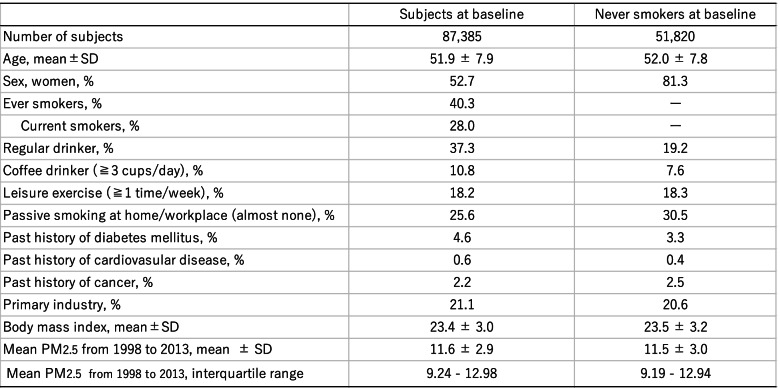
Demogphaphic characteristics of study participants at baseline

**Fig. 1:**
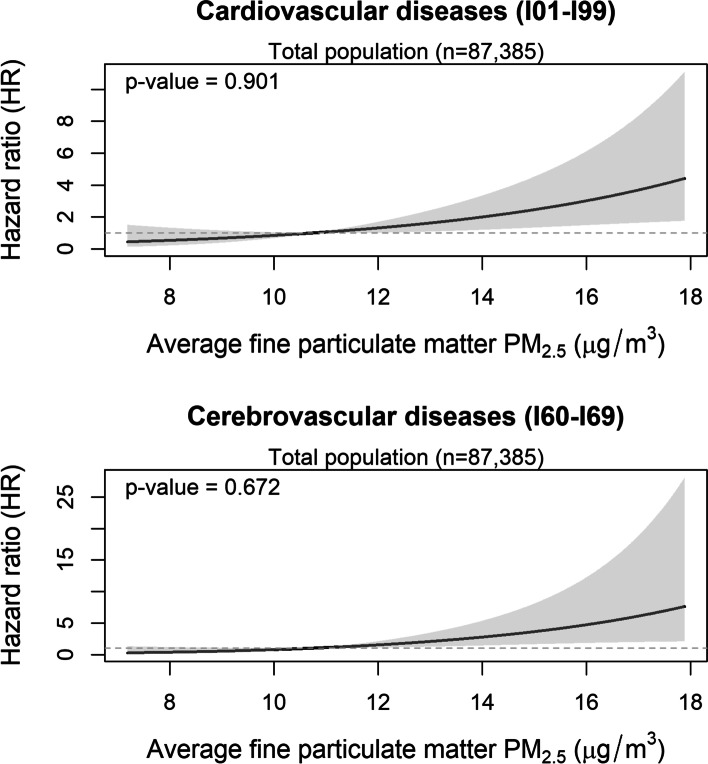
Exposure-response effect of average fine particulate matter PM_2.5_ exposure from 1998 to 2013 on cardiovascular disease mortality (upper panel) and cerebrovascular disease mortality (lower panel) in the whole study population. The curves are based on a restricted cubic spline with a knot placed at the median of the distribution of average PM_2.5_ exposure and the p-values refer to the test of non-linearity of the exposure-response relationship obtained through a likelihood ratio test comparing the depicted spline-based model with a model including only a linear effect of the variable

**Fig. 2:**
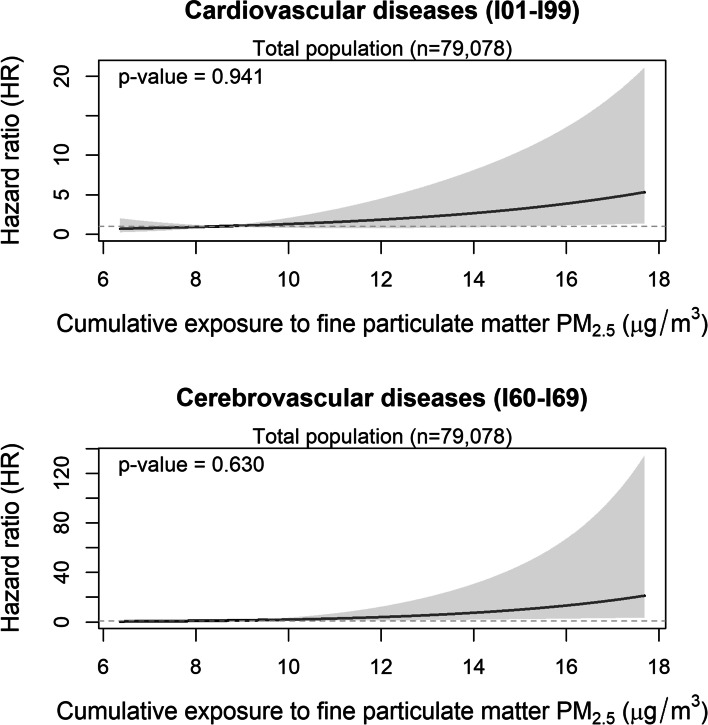
Exposure-response effect of cumulative fine particulate matter PM_2.5_ exposure from 1998 to 2002 on cardiovascular disease mortality (upper panel) and cerebrovascular disease mortality (lower panel) from 2003 onwards in the whole study population. The curves are based on a restricted cubic spline with a knot placed at the median of the distribution of cumulative PM_2.5_ exposure and the p-values refer to the test of non-linearity of the exposure-response relationship obtained through a likelihood ratio test comparing the depicted spline-based model with a model including only a linear effect of the variable

The HRs for all cause and cause-specific mortality are shown in Table 2. PM_2.5_ was not statistically significantly associated with increased risk of all cause mortality; HR for all cause mortality was 1.02 (0.95-1.10). For specific cause mortality, however, PM_2.5_ was positively associated with cardiovascular disease: the multivariate HR of a 1-µg/m^3^ increase in PM_2.5_ for cardiovascular disease mortality was 1.23 (95%CI:1.08-1.40), respectively. In particular, PM_2.5_ exposure was strongly associated with cerebrovascular disease mortality; HRs (95%CI) of a 1 µg/m^3^ increase in PM_2.5_ for cerebrovascular disease mortality was 1.34 (1.11-1.61). Figures 1 and 2 shows the spline-based modelling of the effect of average PM_2.5_ (Fig. 1) and cumulative PM_2.5_ from 1998 to 2002 (Fig. 2) and cardiovascular disease mortality or cerebrovascular disease mortality in the whole study population. In all cases, the relationships monotonously increased with PM_2.5_ exposure level and did not differ significantly from the linear relationships shown in Tables 2 and 3. We did not find associations between PM_2.5_ and other mortality, namely that due to cancer, lung cancer, ischemic diseases, respiratory disease including pneumonia and influenza, or COPD and allied conditions. We also found similar results in never smokers.

**Table 2:**
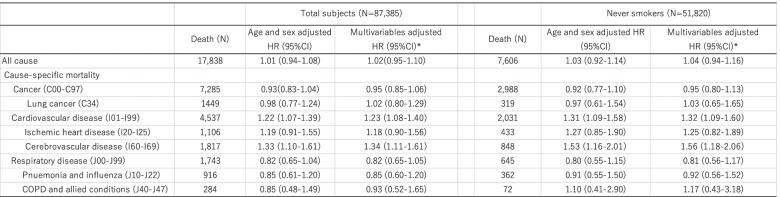
Adjusted HRs for a 1-μg/m3 increase in fine particulate matter PM2.5 (Average from 1998-2013) and 95% CIs for all cause and cause-specific mortality

*Adjusted for age, sex, area (11 area by strata), smoking status (never, past, <20 cigarettes/day, ≧20 cigarettes/day), alcohol drinking (never, occasional, <150g/week, 150-300g/week, ≧300g/week) , body mass index (<18.5, 18.5-23, 23-25, ≧25), occcupation (primary, secondary, tertiary, housewife or unemployed ), coffee intake (<1 cup/day, 1-2 cups/day, 3-4 cups/day, ≧5cups/day), sports (≦3 days/month, 1-4 days/week, almost everyday), passive smoking at home/workplace, and past history of cardiovascular disease, cancer, or diabetes mellitus

**Table 3:**
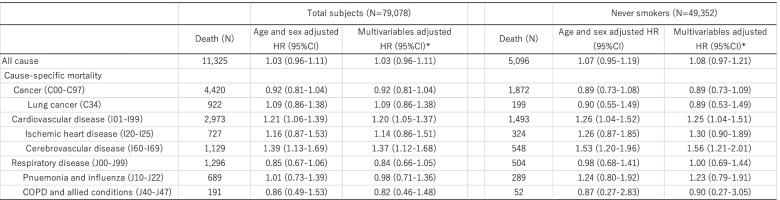
Adjusted HRs for a 1-μg/m3 increase in fine particulate matter PM2.5 (Cumulative average from 1998-2002) and 95% CIs for all cause and cause-specific mortality

*Adjusted for age, sex, area, smoking status (never, past, <20 cigarettes/day, ≧20 cigarettes/day), alcohol drinking (never, occasional, <150g/week, 150-300g/week, ≧300g/week) , body massindex (<18.5, 18.5-23, 23-25, ≧25), occcupation (primary, secondary, tertiary, housewife or unemployed ), coffee intake (<1 cup/day, 1-2 cups/day, 3-4 cups/day, ≧5cups/day), sports (≦3days/month, 1-4days/week, almost everyday), passive smoking (any), and past history of cardiovascular disease, cancer, or diabetes mellitus.

In the sensitivity analysis, to minimize the effects of misclassification due to subjects who censored during follow-up, we used cumulative exposure level before follow-up from 2003 (Table 3). Cumulative average PM_2.5_ exposure (±Standard Deviation) was 10.5 (±3.1) µg/m^3^ and the interquartile range was from 8.28 to 12.68 (Supplemental Table). These results using cumulative 5-year PM_2.5_ data are similar to those using average PM_2.5_ over 15 years.

The results did not change when we adjusted for birth year to control for between-cohort effects.

## Discussion

Overall, the results of this study suggest that PM_2.5_ is not associated with all cause mortality, but that PM_2.5_ is positively associated with cardiovascular disease mortality, especially cerebrovascular disease mortality, even in an area with relatively low exposure.

The positive association between PM_2.5_ and cardiovascular disease mortality is consistent with the results of recent meta-analysis in 2020, which showed the combined effect estimate per 10 µg/m^3^ increase in PM_2.5_ was 1.11 (1.09-1.14) from 21 studies [3]. Possible mechanisms of this increasing risk of cardiovascular disease with PM _2.5_ are considered to involve increasing systemic oxidative stress, endothelial dysfunction and progression of atherosclerosis [18, 27]. Given the results from meta-analysis and mechanisms, these are plausible explanation for our positive association between PM_2.5_ and cerebrovascular diseases, although a recent prospective Japanese cohort reported that PM_2.5_ was not statistically significantly associated with an increase in the risk of mortality due to circulatory disease (I10-I69) nor cerebrovascular disease [15].

Our study showed a larger HR per 1-µg/m^3^ increase in PM_2.5_ for cardiovascular disease (HR=1.23 (95%CI=1.08-1.40)) than a meta-analysis (HR=1.011 (95%CI=1.009-1.014) per 1-µg/m^3^ increase). However, the meta-analysis also showed that summary relative risks (RRs) tended to be larger in studies with mean PM_2.5_ concentrations below 12 or 10 µg/m^3^, for which the corresponding RR per 10-µg/m^3^ increase was 1.12 (1.08-1.17) or 1.17 (1.12-1.23), respectively [3]. Additionally, the “45 and Up Study” of low-level air pollution in Australia showed similar mortality results to ours, with an HR of 1.05 (95%CI=0.98-1.12) per 1-µg/m^3^ increase in PM_2.5_ versus our present HR of 1.02 (95%CI=0.95-1.10), albeit that these researchers did not analyze the risk of cardiovascular disease [28]. Although it is not clear why risk was larger with a lower exposure level than with a high level, our results are nevertheless supported by these previous studies [3, 28].

In our study, the HR of cerebrovascular disease was higher than that of ischemic heart disease. In contrast, a recent meta-analysis reported that the combined effect estimate of ischemic heart disease was slightly greater than that of cerebrovascular disease [3]. This difference in results might be partly explained by the difference in the subtype of cardiovascular disease mortality between Japan and Western countries, wherein mortality of coronary heart disease is lower in East Asian than Western countries and stroke is higher [29].

Outdoor air pollution contains a number of carcinogens, and the International Agency for Research on Cancer (IARC) has concluded that exposure to outdoor air pollution and to particulate matter in outdoor air is carcinogenic to humans (Group 1) and is carcinogenic for lung cancer [16, 17]. A recent meta-analysis of 15 studies also reported a combined effect estimate per 10-µg/m^3^ increase in PM_2.5_ of 1.12 (1.07-1.16) [3]. In contrast, our present study did not show that PM_2.5_ increased risk of lung cancer mortality. We also found that HR was not changed when we limited analysis to never smokers. This finding is not accordant with other Japanese cohorts conducted in one prefecture [15] and three prefectures [30], which reported elevated HRs for lung cancer mortality in association with PM_2.5_. The possibility of a discrepancy might be explained by differences in exposure level. The PM_2.5_levels in these studies were higher than ours: average exposure level (inter-area range) from Katanoda et al. was 16.8 to 41.9 µg/m^3^ [30] while Yorifuji et al. reported an average PM_2.5_level of 14.0 µg/m^3^ [15], which is slightly higher than our average level. It might be difficult to evaluate the adverse effects of air pollution on lung cancer and respiratory disease in areas with a low exposure level. We should also caution about the difference in the number of deaths between cerebrovascular disease and lung cancer: the positive association with cardiovascular disease mortality might have been revealed by the larger number of deaths from cardiovascular disease.

Our JPHC group previously reported that PM_2.5_ was not associated with cardiovascular disease mortality [23], the exposure level for which was assigned using the level at the nearest monitoring station in each public health center area. Rather, our study showed a stronger association between air pollution and cardiovascular disease by using a more accurate method and larger number of cases over longer follow-up, and by excluding subjects who moved residence after baseline. Moreover, our results using cumulative average are similar. According to the European Study of Cohorts for Air Pollution Effects (ESCAPE), which reported a positive association between PM_2.5_ and lung cancer incidence, the ESCAPE study showed a stronger association when restricted to subjects who had lived at the same residence throughout follow-up [31] due to the minimization of misclassification. Our improved evaluation might have identified a clearer association.

The major strengths of this study are its use of a general population with a high response rate (85%) and its prospective design. Additionally, we were able to adjust for possible confounding factors at the individual level. In contrast, several limitations are also present. First, we did not consider exposure level at other places or workplaces due to a lack of information. this raises the possibility of exposure misclassification, because almost no people stay home all day and some are exposed at the workplace, although it is one of best ways identified to date to evaluate individual air pollution exposure level based on their individuals residency. Second, we could not include participants who moved out, because we have not yet applied the geocode to the addresses to which they moved. If a change in residential address is associated with exposure level, this might introduce a bias in the study. Further study using time-varying analysis which combines PM_2.5_ levels at both the original residence and that after moving out among all participants is needed. Third, we did not have information on early exposure, which might have lead to a degree of misclassification. However, we assumed that the spatial PM_2.5_ was preserved in the study area, because average PM_2.5_ (1998-2013) was highly correlated with single year data (0.88-0.99), and because the observed correlation coefficients between concentrations in different years were also high (0.90-0.97). Moreover, the sensitivity analyses which used the cumulative 5yr exposure were robust. We therefore consider that this assumption is reasonable. Fourth, we did not take full account of socioeconomic status, although we adjusted individual job status. Additionally, we adjusted by individual educational level in some subjects whose data were collected by questionnaire, but results were not substantially changed. Fifth, we did not consider change in lifestyle over time, albeit that we did adjust for possible confounding factors at the individual level. Finally, the possibility of residual confounding cannot be ruled out, because confounding factors we adjusted were collected as self-reported.

In conclusion, the present study found evidence that for a positive association between PM_2.5_ exposure and cardiovascular disease mortality in Japanese population, even in a relatively low-level air pollution area.

## Supplementary Information

**Additional file 1.**

## Data Availability

We cannot publicly provide individual data due to participant privacy, according to ethical guidelines in Japan. Additionally, the informed consent we obtained does not include a provision for publicly sharing data. Instructions on how to submit an application to gain access to JPHC data and/or biospecimens are available at https://epi.ncc.go.jp/en/jphc/805/8155.html.
