# Supplementary material for: Long-term exposure to fine particle matter and all-cause mortality and cause-specific mortality in Japan: the JPHC Study
Source: BMC Public Health. 2022 Mar 8;22:466. doi: 10.1186/s12889-022-12829-2 (PMC8905772; doi:10.1186/s12889-022-12829-2)
Supplement: Supplementary file 1 — Additional file 1. [file 12889_2022_12829_MOESM1_ESM.pdf]

SupplementTable. Demogphaphic characteristics of study participants from 2003.		
	Subjects at 2003	Never smokers at 2003
Number of subjects	79,078	49,352
Age, mean ± SD	63.1 ± 7.5	63.2 ± 7.4
Sex, women, %	53.6	78.9
Ever smokers, %	37.6	—
Current smokers, %	22.5	—
Regular drinker, %	43.6	28.0
Coffee deinker (≧3 cup/day), %	22.1	17.7
Leisure exercise (≧1 time/week), %	22.7	24.1
Passive smoking at home/workplace (almost r	13.2	17.7
Past history of diabetes mellitus, %	6.4	5.2
Past history of cardiovasular disease, %	3.0	2.4
Past history of cancer, %	4.7	4.5
Primary industry, %	21.7	22.5
Body mass index, mean ± SD	23.6 ± 3.2	23.7 ± 3.2
Cumulative average $PM_{2.5}$ from 1998 to 2012, mean ± SD	10.5 ± 3.1	10.3 ± 3.2
Cumularive average PM2.5 from 1998 to 2012, interquartile range	8.28 - 12.68	8.24 - 12.64
